# Supplementary figures and images for: B-Cell Activating Factor Enhances Hepatocyte-Driven Angiogenesis via B-Cell CLL/Lymphoma 10/Nuclear Factor-KappaB Signaling during Liver Regeneration
Source: Int J Mol Sci. 2019 Oct 10;20(20):5022. doi: 10.3390/ijms20205022 (PMC6829427; doi:10.3390/ijms20205022)

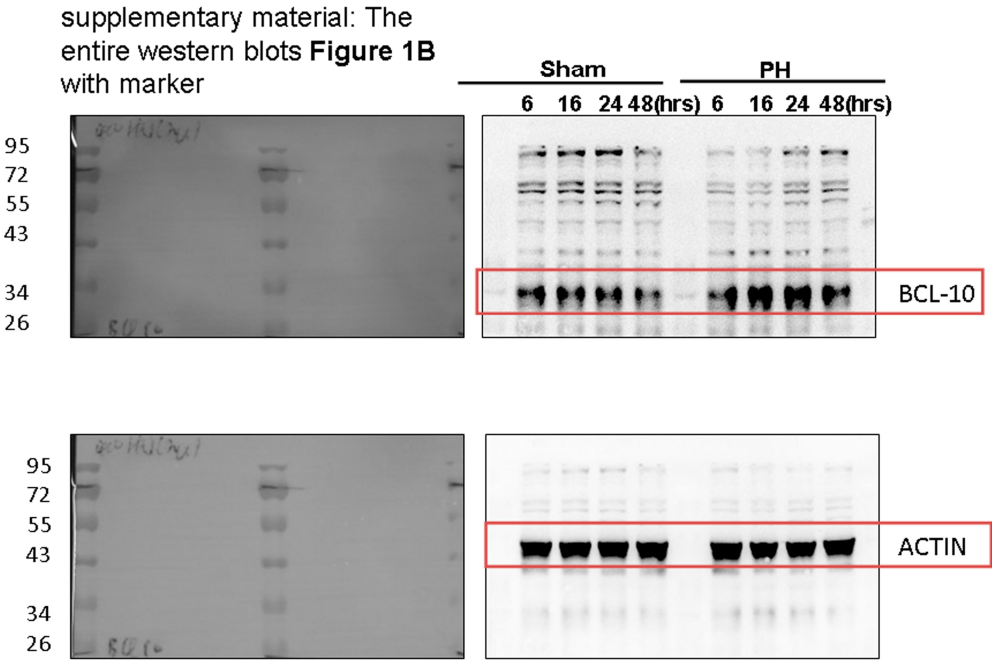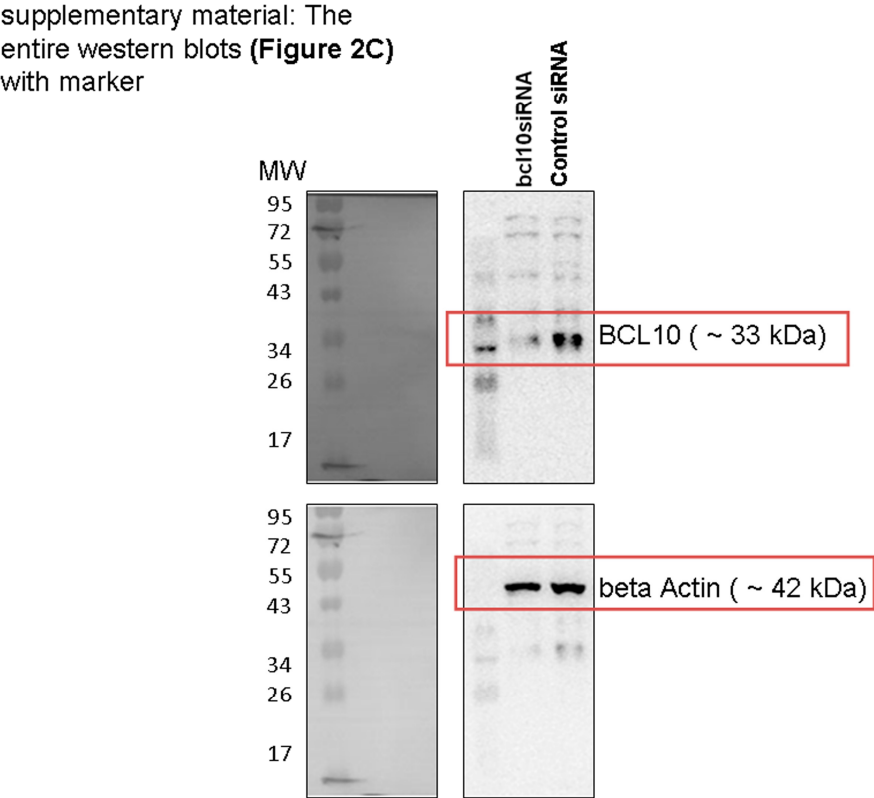

Supplement: Supplementary file 1 [file ijms-20-05022-s001.pdf]
